# Supplementary figures and images for: Kinetics of HTLV-1 reactivation from latency quantified by single-molecule RNA FISH and stochastic modelling
Source: PLoS Pathog. 2019 Nov 18;15(11):e1008164. doi: 10.1371/journal.ppat.1008164 (PMC6886867; doi:10.1371/journal.ppat.1008164)

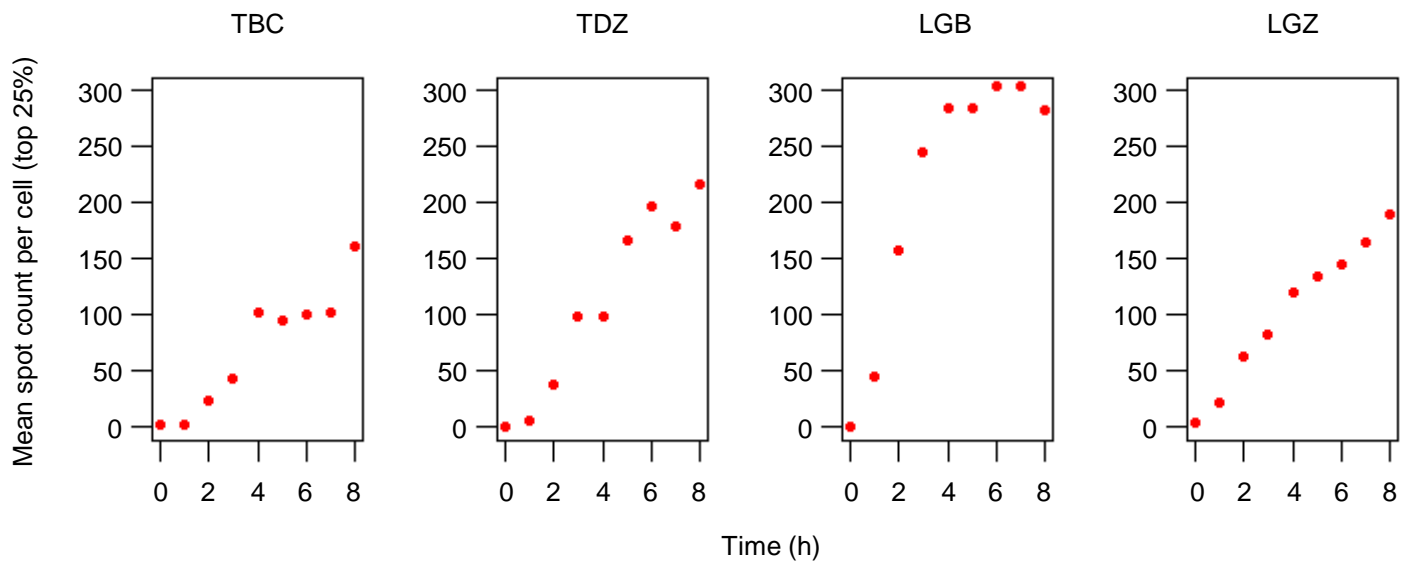

Supplement: S1 Fig — The average number of HTLV-1 sense transcripts in the top 25% of all the infected cells at successive timepoints during in vitro incubation. The data from two patients with HAM (TBC and TDZ) and two with ATL (LGZ and LGB) are presented. (PDF) [file ppat.1008164.s001.pdf]

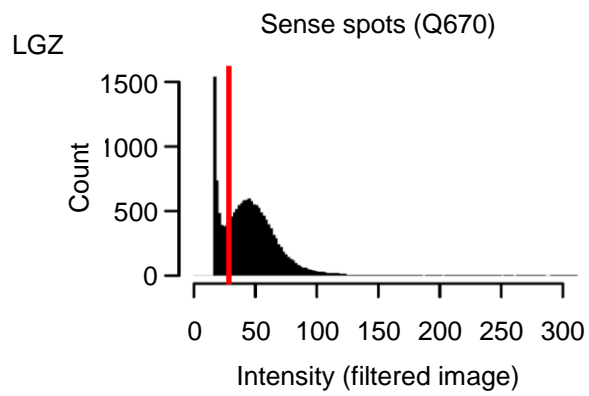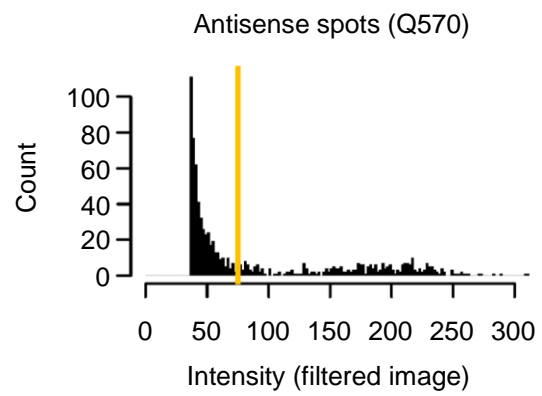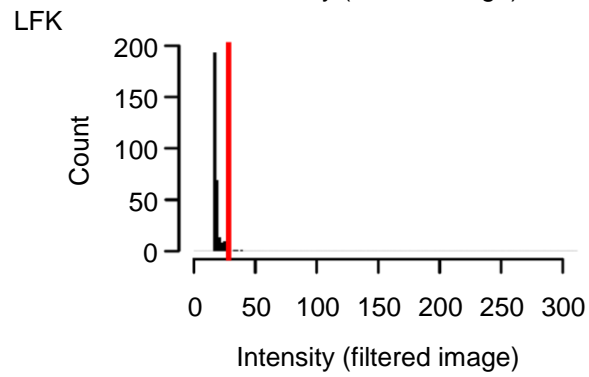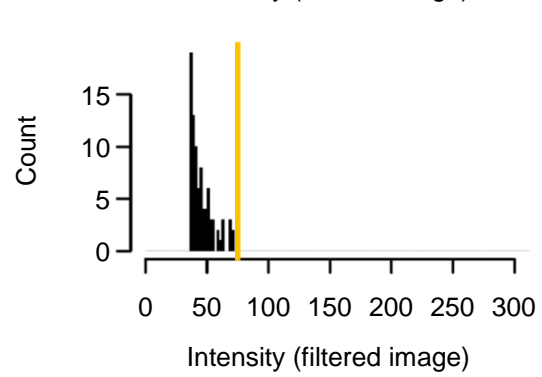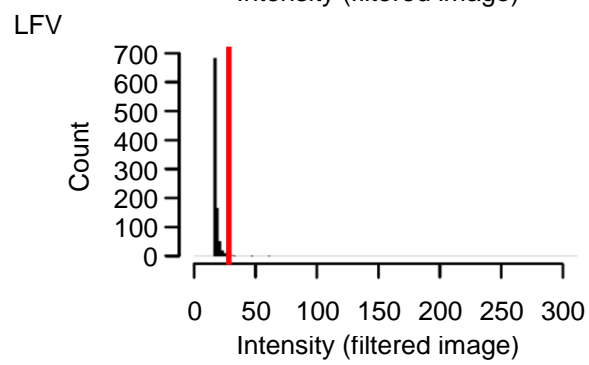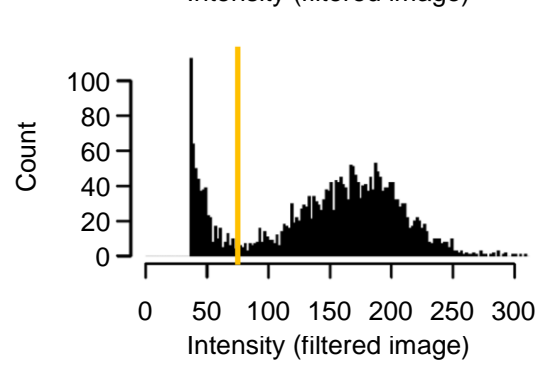

Supplement: S2 Fig — Histograms of the intensity of spots pre-detected with FISH-QUANT [1] with a conservative threshold setting. The first column shows the spot intensity for Q670, and the second column for Q570. For the analysis, a more stringent threshold was set for each of the channels (red or yellow vertical lines) to remove the spurious signals. The samples shown in the panel (LGZ incubated and fixed at 7 h, LFK and LFV fixed without incubation) were hybridized and imaged in a single batch. (PDF) [file ppat.1008164.s002.pdf]

Model (ii)

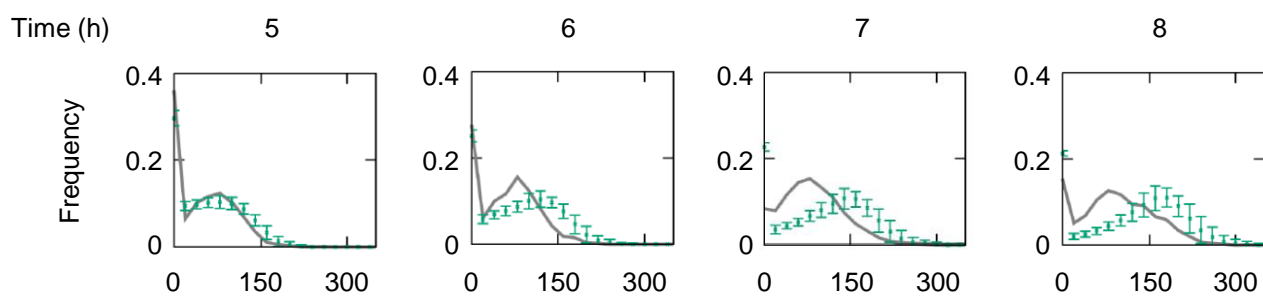

Model (iii)

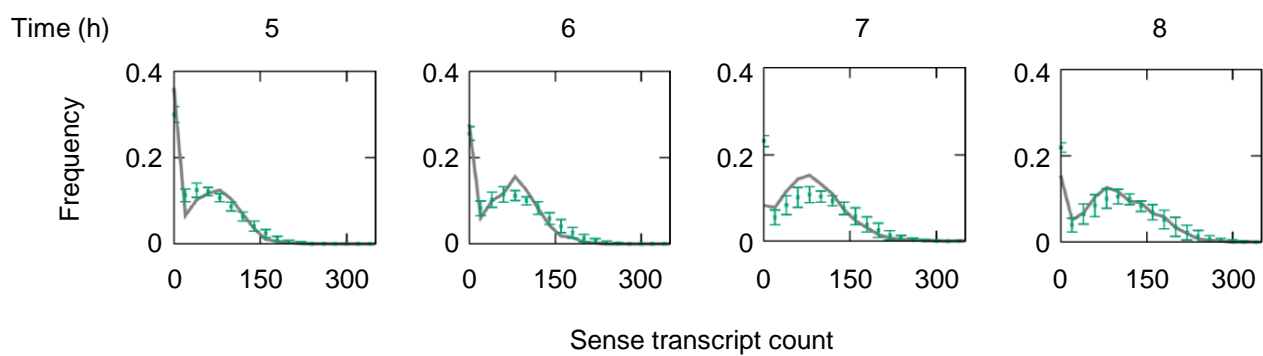

Supplement: S3 Fig — To test the performance of the models, we used the experimental data (LGZ) from T = 1, 2, 3 and 4 hrs (omitting the data from T = 5, 6, 7 and 8 hrs) to estimate the best 50 parameter sets from 106 iterations of the models. We used the resulting 50 parameter sets to predict the data points at T = 5, 6, 7 and 8 hrs that were omitted from the parameter estimation. The mean predicted frequency and its standard deviation in each bin are plotted in green and overlaid on the experimental observation (solid grey line). (PDF) [file ppat.1008164.s003.pdf]

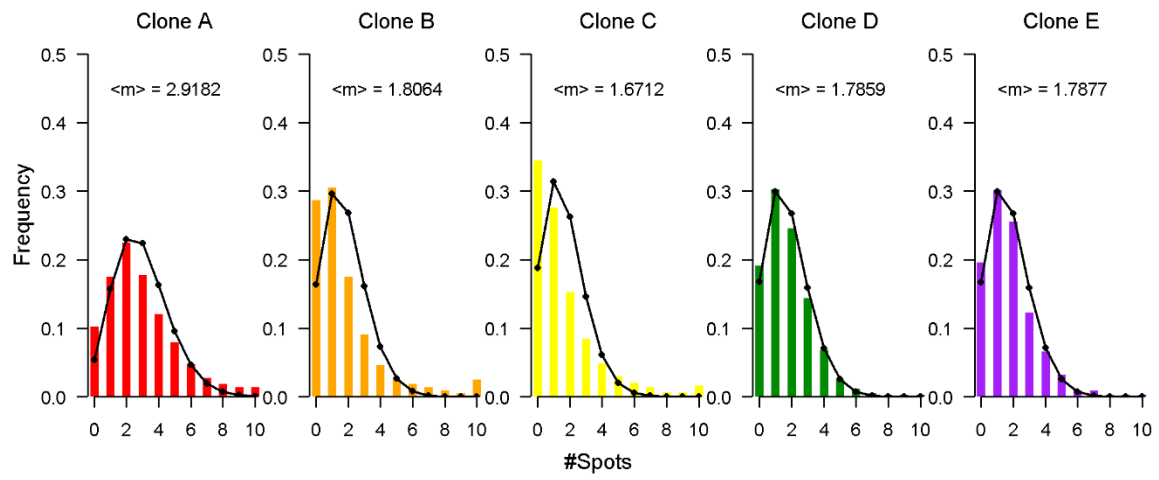

Supplement: S5 Fig — The distribution of the number of HBZ molecules per cell in in vitro maintained HTLV-1+ T cell clones. Histograms are reproduced from the graphs presented in Billman et al 2017 [3]. The mean number of HBZ molecules () is indicated in the inset. (The last bin is indicated as 10 or more spots in the original paper (Ref 1); in the present figure the bin is shown as = 10 spots). The black line indicates the Poisson distribution with the parameter , the observed mean number of HBZ molecules. (PDF) [file ppat.1008164.s005.pdf]

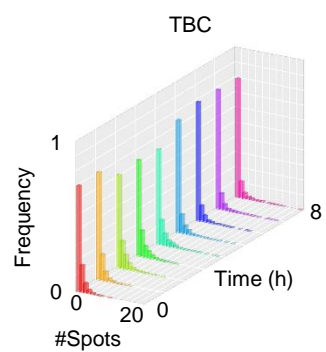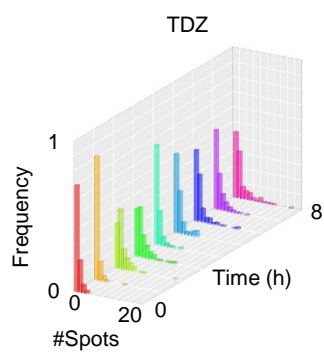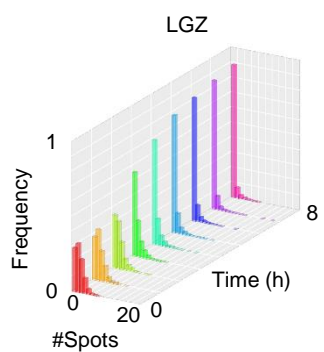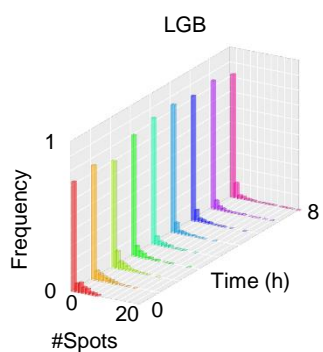

Supplement: S6 Fig — HBZ mRNA count per HTLV-1+ cell at successive timepoints during in vitro incubation. The data from two patients with HAM (TBC and TDZ) and two with ATL (LGZ and LGB) are presented. (PDF) [file ppat.1008164.s006.pdf]

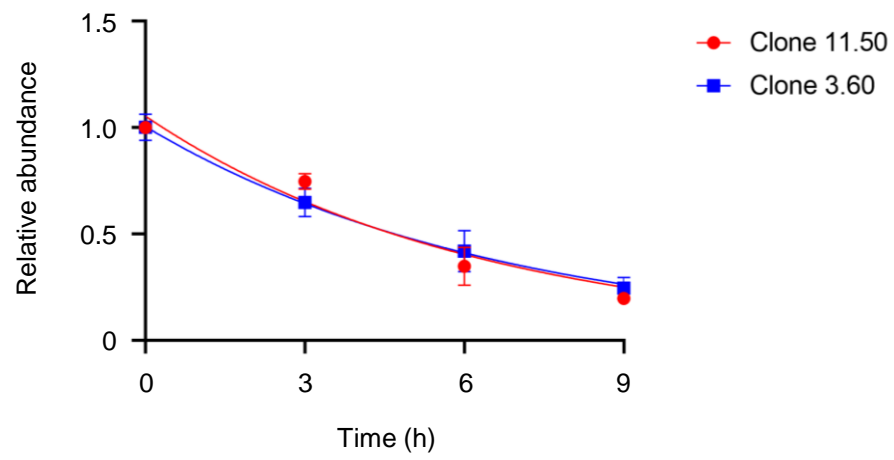

Supplement: S7 Fig — Two in vitro HTLV-1-infected T cell clones were treated with actinomycin D to block transcription, and the abundance of HTLV-1 sense-strand transcripts was quantified at each time point (0, 3, 6 and 9 hours) (see Materials and methods). The error bars indicate the standard deviation of the qPCR technical replicates. The half-life of the HTLV-1 sense-strand transcripts was estimated to be 4.33 h (Clone 11.50) and 4.66 h (Clone 3.60). The combined estimation of the half-life was 4.45 h, with the 95% confidence interval of 2.70–5.61 h. (PDF) [file ppat.1008164.s007.pdf]
