# Supplementary material for: Kinetics of HTLV-1 reactivation from latency quantified by single-molecule RNA FISH and stochastic modelling
Source: PLoS Pathog. 2019 Nov 18;15(11):e1008164. doi: 10.1371/journal.ppat.1008164 (PMC6886867; doi:10.1371/journal.ppat.1008164)

A

Reaction	Parameter	Description	Parameter range (h^{-1})
Transition	k_1	Off to On	0.1 - 10
	k_2	On to Off	0.1 - 100
	k_3	On to On-Tax	0.1 - 10
	k_4	On-Tax to On	0.1 - 100
	k_{10}	On-Tax to Off	0.01 - 10
Transcription	k_5	From On state	0.1 - 25
	k_6	From On-Tax state	10 - 100
Translation	k_8	Tax translation	0.01 - 10
Degradation	k_7	Sense mRNA	0.154 (fixed)
	k_9	Tax protein	0.105 (fixed) (Ref 2)

B

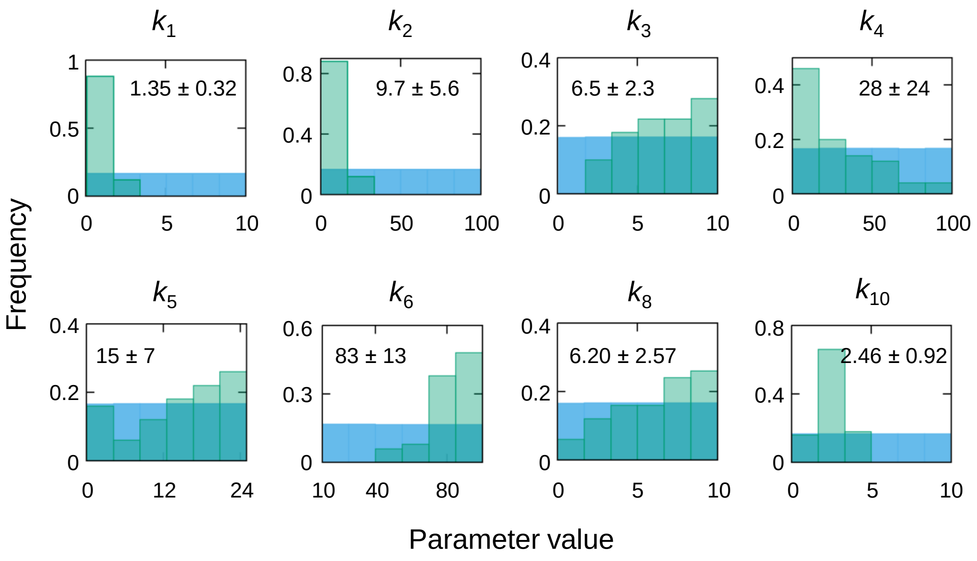

Supplement: S4 Fig — (A) To check the parameter convergence, we repeated the parameter search within a second set of prior distributions. (B) We performed 106 iterations with the above prior set and selected the best 50 parameter sets with the minimum distance function (see also S1 Text and [2]). The histograms depict the distributions of 50 parameters (green bars) for each reaction in the model; the respective prior distributions are shown in blue. The mean and the standard deviation are indicated in the inset. (PDF) [file ppat.1008164.s004.pdf]
