# Supplementary material for: Kinetics of HTLV-1 reactivation from latency quantified by single-molecule RNA FISH and stochastic modelling
Source: PLoS Pathog. 2019 Nov 18;15(11):e1008164. doi: 10.1371/journal.ppat.1008164 (PMC6886867; doi:10.1371/journal.ppat.1008164)
Supplement: S2 Text — (PDF) [file ppat.1008164.s009.pdf]

An alternative model accounting for the departure from the Poisson distribution

We observed a deviation in the *HBZ* count per cell in some of the *in vitro* maintained HTLV-1^{+} T cell clones from the value calculated from the Poisson distribution (Ref 1). Specifically, there was a greater number of *HBZ*-negative cells in each clone at a given time than predicted by the Poisson distribution. We suspected that this deviation was due to the transcription of the antisense strand in small and occasional bursts. Therefore, we applied the following two-state model to clone B as an example.

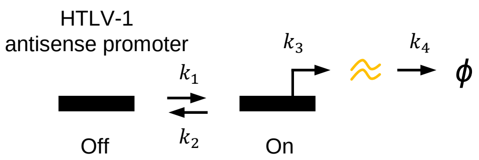

The number of *HBZ* molecules <m> at the steady state can be described as

$$< m > = \frac{k_3[On]}{k_4} = \frac{k_3}{k_4} \cdot \frac{k_1}{k_1 + k_2}$$

We estimated the degradation rate k_4 to be 0.1575 h^{-1} as previously reported from experimental measurements (Ref 1). We carried out the parameter search for k_1 and k_3 within the range of 0.001 to 10 and 0.05 to 50, respectively. The parameter k_2 was constrained by

$$k_2 = \frac{k_1 k_3}{< m > \cdot k_4} - k_1$$

We obtained a parameter set (k_1=0.658, k_2=94.524, k_3=41.14 and k_4=0.158) which yielded the distribution indicated below by the black line.

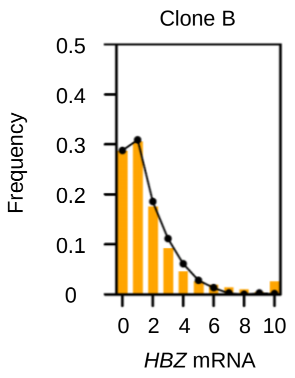

With this parameter set, we estimate the burst frequency and intensity as follows:

burst frequency = k_1[Off] = $k_1 \cdot k_2$ / (k_1+k_2) ~ 0.653 (h^{-1})

burst intensity = k_3 / k_2 ~ 0.435 (molecules / burst)

Reference

1. Billman MR, Rueda D, Bangham CRM. Single-cell heterogeneity and cell-cycle-related viral gene bursts in the human leukaemia virus HTLV-1. Wellcome Open Res. 2017;2:87.
