# Supplementary material for: Kinetics of HTLV-1 reactivation from latency quantified by single-molecule RNA FISH and stochastic modelling
Source: PLoS Pathog. 2019 Nov 18;15(11):e1008164. doi: 10.1371/journal.ppat.1008164 (PMC6886867; doi:10.1371/journal.ppat.1008164)
Supplement: S1 References — (DOCX) [file ppat.1008164.s010.docx]

Supporting Information: references

1. Mueller F, Senecal A, Tantale K, Marie-Nelly H, Ly N, Collin O, et al. FISH-QUANT: automatic counting of transcripts in 3D FISH images. Nat Methods. 2013;10(4):277-8.

2. Rende F, Cavallari I, Corradin A, Silic-Benussi M, Toulza F, Toffolo GM, Tanaka Y, Jacobson S, Taylor GP, D'Agostino DM, Bangham CR, Ciminale V. Kinetics and intracellular compartmentalization of HTLV-1 gene expression: nuclear retention of HBZ mRNAs. Blood. 2011 May 5;117(18):4855-9.

3. Billman MR, Rueda D, Bangham CRM. Single-cell heterogeneity and cell-cycle-related viral gene bursts in the human leukaemia virus HTLV-1. Wellcome Open Res. 2017;2:87.
